# Notes and Formulæ

**Published:** 1889-12

**Authors:** 


### PRACTICAL NOTES AND FORMULÆ.

---

**Cholera Infantum.**—I send formula for cholera infantum that I have used for fifteen years with unvarying success. I claim no originality for the formula, but simply publish it for the benefit of the professsion, knowing it to be thoroughly reliable.

| | | |
|---|---|---|
| ℞ | Tinct. nucis vom. | 2 drachms. |
| | Nitro muriatic acid | 2 drachms. |
| | Bismuth subnit. | 2 drachms. |
| | Lactopeptine, or Jensen's pepsin | 2 drachms. |
| | Syr. simp. | 4 ounces. |
| | Sherry wine | 4 ounces. |

M. Sig. For child of one and one-half years old, one teaspoonful in a little water; others in proportion to age, three or four times a day.—W. S. Randolph, M. D. in *Med. Brief.*

**Chronic Rheumatism.**—To Dr. C. C. Rutherford, page 111, would suggest a trial in his case of chronic rheumatism of the following prescription, from which I have derived much satisfaction in like cases, viz.:

| | | |
|---|---|---|
| ℞ | Potass iod. | 4 drachms. |
| | Tinct. opii | 4 drachms. |
| | Ext. manaca fl. | 4 drachms. |
| | Ext. colchicum fl. | 4 drachms. |
| | Syr. yerba santæ, q. s. | 4 ounces. |

M. Sig. One teaspoonful every six hours.—*Ib.*

**Thiol.**—Thiol can be used with gratifying results in many forms of skin diseases, notably in:

Acne, eczema, erysipelas, leprosy, burns, scalds and frost bites.

The drug is of such great value that I give complete formulæ and trust all will give it a trial.

| | | |
|---|---|---|
| ℞ | Thiol | 2 grains. |
| | Pulv. glycyrrhiza | 2 grains. |
| | Glycerine | q. s. |

M. ft. pil. 8 Sig. One three times a day.

| | | |
|---|---|---|
| ℞ | Thiol | 1 drachm. |
| | Zinci oxide | 2 drachms. |
| | Amylum | 1 drachm. |
| | Talc. | 2 drachms. |

M. Sig. Dust on parts at night.—*Ib.*

**Gastralgia.**—

| | |
|---|---|
| ℞ Bismuth subnit. | 4 drachms. |
| Tinct. nucis vom. | 48 drops. |
| Fowler's solution | 90 drops. |
| Aquæ | 2 ounces. |

M. Sig. One teaspoonful after each meal. Shake well before using.—*Ib.*

**Nasal Catarrh.**—Prof. Leffert's solution for nasal catarrh is as follows:

| | |
|---|---|
| ℞ Acidi carbolici | 1 ℈ |
| Sodii boratis | 1 ʒ |
| Sodii bicarbonatis | 1 ʒ |
| Glycerine | 1 ℥ |
| Aquæ rosæ | 1 ℥ |
| Aquæ, q. s., ad | oi |

DEODORIZED IODOFORM OINTMENT—

| | |
|---|---|
| ℞ Iodoform in powder | ——— |
| Parched coffee, in powder, of each | 3 grms |
| Vaseline | 30 grms |

—*Gazetta Medica di Tarino.*

As a vaginal wash in vaginitis:

| | |
|---|---|
| ℞ Creolin | ʒj |
| Aquæ | f℥xvj |

—Prof. Parvin in *Col. and Clin. Rec.*

For a man of 46 with bronchial neuralgia:

| | |
|---|---|
| ℞ Atropinæ sulph., | gr. $\frac{1}{80}$ |
| Morphinæ sulph., | gr. $\frac{1}{6}$ |

Sig. Hypodermically once a day.
Also, small blisters over points of redness.

—Prof. daCosta in *Clin. Rec.*

**Goitre.**—Prof. daCosta brought before the clinic a woman with goitre, and prescribed:

| | |
|---|---|
| ℞ Iodinii, | gr. xxx |
| Lanolin, | ℥j |
| Ol. juniperi, | gtt. v M |

Fiant unguentum.

Sig. Apply over the goitre. Also three drops of Churchill's tinc. of iodine, well diluted, to be taken three times a day.—*Clin. Rec.*

**Treatment of Asthma.**—We occasionally meet cases of continued distress despite the use of ordinary means. In such cases there is usually much bronchial tumefaction and dryness. In cases of this class nothing equals ¼ gr. pilocarpine with ¼ gr. morphine, administered hypodermically. Relief is prompt, tumefaction subsides, and is followed by profuse expectoration.—*Clin. Rec.*

